# Chronic Pain Self-Management Strategies for Older Adults: An Integrative Review

**DOI:** 10.3390/life14060707

**Published:** 2024-05-30

**Authors:** Thaiany Pedrozo Campos Antunes, Fernanda Golçalves Jardim, Cláudia Inês Pelegrini de Oliveira Abreu, Luiz Carlos de Abreu, Italla Maria Pinheiro Bezerra

**Affiliations:** 1 Public Policy and Local Development Department, Superior School of Sciences of the Santa Casa de Misericórdia de Vitória, Vitória 29045-402, Espirito Santo, Brazil; thaianycampos@yahoo.com.br (T.P.C.A.); nandajardim2013@hotmail.com (F.G.J.); luiz.abreu@ufes.br (L.C.d.A.); 2 Department of Preventive Medicine, Federal University of Espirito Santo, Vitória 29075-910, Espírito Santo, Brazil; claudiainespabreu@gmail.com

**Keywords:** geriatric pain, aging, chronic pain, self-management, pain coping

## Abstract

Introduction: Due to the complex nature of chronic pain, especially in older adults, a biopsychosocial approach is more effective than an isolated approach for its management. Furthermore, when patients are actively engaged in their pain management, they are more likely to be successful than relying totally on others. Objective: To analyze the self-management strategies currently used by older adults with chronic pain. Method: An integrative review was conducted through seven online databases, searching for scientific studies on this topic published in the last 10 years. Results and conclusion: Fifty-eight studies were included in the final sample. Research on chronic pain self-management for older adults has increased in recent years. Although a diversity of chronic physical painful conditions are being investigated, many conditions are still under-investigated. Online and in-person strategies are currently adopted, demonstrating similar results. Positive results are evidenced by strategies including health promotion, mind control, social participation and take-action fields. Major results come from a combination of strategies focusing on biopsychosocial aspects of pain management. Results include not only the reduction of pain itself, but increased self-efficacy, adoption of health behaviors and improvement of functionality, among others, i.e., improved QoL, despite pain.

## 1. Introduction

People aged 60 and older represent the fastest-growing age group today. It is estimated that by 2050, one in six people in the world will be over the age of 65 (16%), compared to one in eleven people (9%) in 2019 [1]. The incidence of many diseases increases with aging, leading to various dysfunctions [2] that are related to or recurrent from pain, such as previous traumatic injuries, physiological changes in the connective tissue related to aging, degeneration of the nervous system [3], musculoskeletal diseases [4], cancer [5] and diabetes [6].

The prevalence of chronic pain increases significantly with advancing age [7]. The prevalence of pain in the elderly, especially chronic pain, is quite high, estimated at up to 85%, compared to a group of younger adults, estimated at 7.3–68% in the 40–66 age group [8]. Still, it is possible that conformational attitudes of the elderly, towards beliefs about pain being a natural element of aging and fears about a potential pharmacological addiction, may lead to underreporting and undertreatment of pain in this population [9], with functional impairment and decreased quality of life [10].

Chronic pain is difficult to accurately diagnose and treat due to its complex and multifactorial nature. The cost of pain to society is high, not only in financial terms, but also in terms of physical and emotional suffering, including impaired cognitive function, depression, sleep disturbance, impaired socialization and impaired skills [11]. Older adults are particularly more difficult to treat [12] due to the high prevalence of multiple chronic painful conditions in this population, such as degeneration in bones, joints and muscles [13], and the use of polypharmacy, including opioid overuse, with the possibility of drug interactions and side effects [14]. Therefore, the management of geriatric pain should consider its impact on activities of daily living and patient independence [15].

To produce the best effects not only on pain but also on function, a multimodal approach is required. Studies have indicated that a multidisciplinary and biopsychosocial approach is more effective than an isolated approach for the treatment and management of chronic pain [16]. A pain management plan is more likely to be successful when patients are actively engaged in multiple modalities related to pain management [17].

Therefore, many studies focused on self-management of chronic pain in older adults were found, demonstrating the need for understanding of this patient cohort regarding the neurophysiology of pain and the influence of emotional symptoms (stress, anxiety, kinesiophobia, etc.), lack of sleep and physical activity in chronic pain, encouraging gradual exposure to natural movements and physical activity for pain self-management [18]. However, these studies have been limited to one or a few specific painful body regions, such as low back pain [18], neck pain, or knee pain [19], or to a baseline condition in which pain is one of the symptoms, such as osteoarthritis [20] or fibromyalgia [21]. Studies on the self-management of chronic pain as a disease in older adults in a more comprehensive way were not found. Thus, this study aims to identify the self-management strategies currently used by older adults with chronic pain and evaluate their effectiveness for managing chronic pain symptoms in this population.

## 2. Method

An integrative review was conducted in five stages: (1) problem identification, with research question definition; (2) literature search; (3) data evaluation, with studies’ categorization; (4) data analysis and (5) presentation of findings [22]. An integrative review is a method that summarizes the empirical or theoretical literature to provide a greater comprehensive understanding of concepts, theories, evidence of gaps in the literature and methodological issues [23].

To elaborate the research question, the PICo strategy was applied (P—population, context and/or problem situation; I—intervention; Co—condition). Thus, the following structure was considered: P—older adults; I—self-management strategies; Co—chronic pain. In this way, the following guiding question was elaborated: “What are the self-management strategies currently used by older adults with chronic pain?”

The initial search was carried out on 19 October 2022, through seven online databases: Medline (via PubMed, www.pubmed.com (accessed on 18 October 2022), Scopus and Web of Science (via CAPES—Coordination for the Improvement of Higher Education Personnel, www.periodicos.capes.gov.br (accessed on 20 October 2022), International Association for the Study of Pain via IASP, www.iasp-pain.org (accessed on 20 October 2022), Physiotherapy Evidence Database, via PEDro, https://pedro.org.au/ (accessed on 20 October 2022), Brasil Scientific Eletronic Library Online, via SciELO, www.scielo.br (accessed on 18 October 2022) and Literatura Latino-Americana e do Caribe em Ciências da Saúde, via LILACS, https://lilacs.bvsalud.org/en/ (accessed on 18 October 2022). Keywords and Mesh terms were used, merging them through Booleans “AND” and “OR”; therefore, the search strategy was mostly as follows: (elderly OR “older adult*” OR “senior*”) AND (education OR educational) AND (“self-care” OR selfcare OR “self care” OR coping OR “self control” OR “self-control” OR “self-management” OR “self management”) AND (pain OR ache OR “physical suffering”). On PubMed, terms were searched when included in titles or abstracts; on Scopus, when included in the title, abstract or keywords; on Web of Science, when included in the topic; and on LILACS, when included in the title, abstract or subject. On IASP, articles on drug development, animal studies, itch and basic neurobiology were excluded, and on PEDro, which has a different search strategy, the advanced search tool was used, selecting the following terms: education, for “therapy”; pain, for “problem”, gerontology, for “subdiscipline” and chronic pain, for “topic”.

Inclusion criteria encompassed studies using self-management strategies, including both clinical trials or observational studies, not only specifically for people aged 60 years or more, but including this population, of both genders, with primary or secondary chronic pain. Exclusion criteria encompassed studies that did not cover self-management strategies (i.e., passive, invasive, drug, electrotherapy or group interventions); studies on animals or other populations (health professionals, caregivers or people of other age groups); studies not related to the topic (i.e., studies about acute pain, exclusively of diagnostic and evaluation methods, sample characterization, risk factors, association of variables not related to pain management, validation of instruments, about anatomical or physiological alterations); protocol, pilot and review articles; articles in languages other than English, Portuguese or Spanish; articles that are not fully available for free; and studies older than 10 years (published before 2012).

For study selection, duplicated articles were excluded. Then, titles and abstracts were screened by the theme. This selection was based on studies about self-management for chronic pain, not exclusively of older adults but including older adults.

To increase confidence in the selection of articles, all search and selection stages were reviewed independently by at least two researchers who, after reading all potential articles, reached a consensus to establish which articles met the inclusion criteria [24]. Discrepancies were resolved by discussion between the two review authors. There was no discrepancy that required the decision of a third author.

## 3. Results

**Figure 1:**
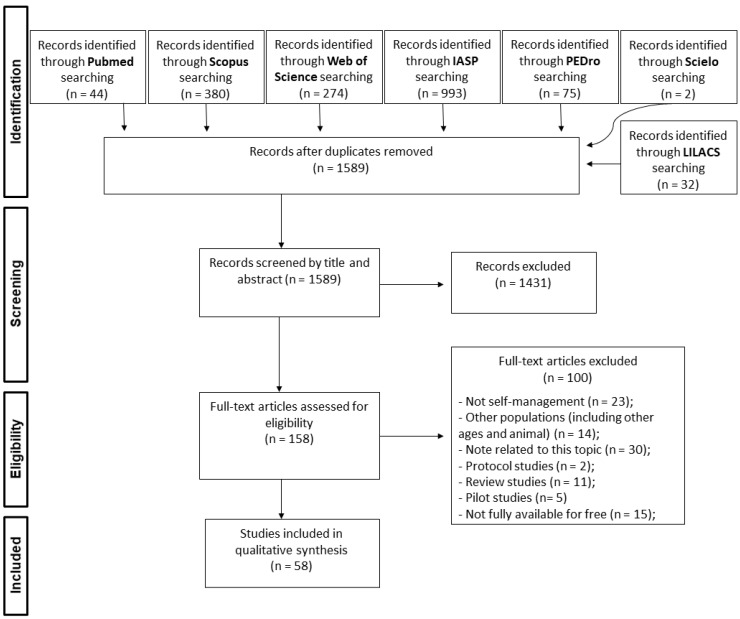
Flowchart of article selection.

A total of 1800 papers were found in the initial search in all databases. After deleting duplicate papers, 1589 were filtered by title and abstract, from which 58 papers were included in the final sample of this integrative review (Figure 1).

The articles included in this study were organized in a digital table, numbered in ascending order, according to the date of publication, with article number 1 being the oldest. This digital document also contained the following information about each of the articles: author, year, hyperlink to find it in its online version, painful condition addressed in the article, self-management strategy used, objective, summary of the method and main findings.

During the observed period, publications on this topic increased up to 2020, with a decline in 2021 and in 2022. In 2012, there was only one paper published on this topic; then, three were published in 2013 and 2014, six in 2015, seven in 2016, 2017 and 2019, five in 2018, and 10 in 2020. In 2021, six papers were published, and two papers were published up to October 2022, when the initial search was conducted.

The included studies investigated a diversity of chronic physical painful conditions, and some of them investigated multiple conditions. The most prevalent medical condition investigated was chronic pain (CP) (33%) itself, followed by low back pain (LBP) (21%) and osteoarthrosis (OA) in various regions (hip, knee, general) (21%), other chronic diseases (7%), rheumatoid arthritis (RA) (5%), and back (5%) and knee pain (5%). Other diseases were also investigated, but only in one study of each, among them: hip pain, neck pain, lower extremity pain, lumbar spinal stenosis (LSS), spondylitis, fibromyalgia, irritable bowel syndrome (IBS) and migraine. 

Regarding self-management strategies adopted, both online [20,25,26,27,28,29,30,31,32,33] and in-person strategies were investigated. Most of studies combined more than one strategy or investigated more than one strategy in different groups. The most-investigated strategy was the individual or group self-management course/program, investigated in 52% of the studies [19,20,25,26,27,28,29,30,31,33,34,35,36,37,38,39,40,41,42,43,44,45,46,47,48,49,50,51], which encompassed a mix of the remaining strategies. Following, the order of the most-investigated strategies was physical exercise (in 34% of the studies), that could be specific [32,52,53,54,55] or general exercises [20,21,25,35,48,49,56,57,58,59,60,61,62,63,64], individually or in groups, followed by education (in 21% of the studies) [21,32,36,52,53,54,56,58,60,63,64], cognitive behavioral therapy (19% of the studies) [29,34,39,47,51,55,64,65,66,67,68], meditation/auto-hypnosis (7%) [69,70,71], self-monitoring [57,72,73], booklet [56,59,74], lay-peer strategies [49,50,75] and activity pacing [34,48,76], representing 5% of the studies each, diet (4%) [44,77] and self-created coping strategies (2%) [78]. 

Self-management strategies was divided into four fields (Table 1): (1) Heath promotion, covering education on pain, its causes and triggers, the main methods of diagnosis and treatments and behavior and life-style change; (2) Mind control, which encompasses knowledge about oneself, one’s thoughts and faith; (3) Social aspects, motivating older patients to share their experiences of chronic pain to teach and learn about it and to help others (through lay-peer strategies, for example); and (4) How to do, which includes strategies to execute the appropriate physical exercise, self-efficacy, that is the ability to organize and act to reach clinical objectives, and self-monitoring, to ensure continuity of results. Table 1 presents the general topic and contents covered by the main strategies investigated. 

Most studies investigated more than one dependent variable, and most of them presented positive outcomes, yielding improvement of many symptoms after the practice of the chosen pain self-management strategy (Table 2). The most evidenced positive outcome was related to pain itself (51.72%) [20,25,28,29,30,32,33,35,36,38,40,43,44,45,47,48,49,53,54,55,58,59,61,63,64,65,66,67,68,69,70,74,75,78,79]. Self-strategies investigated resulted in the reduction of pain intensity and/or the number of spots presenting pain. In second place were increased self-care, self-efficacy, self-management and/or health behaviors (24.14%) [26,33,36,37,39,42,45,51,53,54,55,56,57,59,73,79], followed by increased physical function (20.68%) [19,20,21,28,34,36,40,42,45,52,56,58,61,62,73] and reduced depression (18.96%) [26,29,30,32,35,37,39,49,66,68,70]. Table 2 presents some examples of positive findings of the studies included, organized according to the main aspect addressed by their outcomes: biological, psychological, social or a mix of them. The majority of outcomes addressed biological aspects, followed by psychological aspects.

Some studies (13.8%) also presented negative or neutral outcomes from the adopted pain self-management strategy, as shown in Table 3.

## 4. Discussion

Due to the complex nature of chronic pain, especially in older adults, a biopsychosocial approach is more effective than an isolated approach for its management [16]. Furthermore, when patients are actively engaged in their pain management, they are more likely to be successful than when relying totally on others [17]. Therefore, this integrative review aimed to assemble the self-management strategies currently used by older adults with chronic pain.

It was possible to observe that from 2012 on, the number of studies progressively increased, except in the years 2021 and 2022. This lack of continuity in publications on this theme was probably related to the COVID-19 pandemic, after the interruption of many fields of study due to physical isolation recommended by the World Health Organization, while research on COVID-19 effects intensified [80].

Due to psychological and physiological changes common to most chronic pain conditions, such as emotional distress and/or functional disability [81], a diversity of chronic physical painful conditions was investigated in the studies included in this integrative review. The most prevalent disease investigated was CP, followed by LBP and OA. Indeed, these are painful conditions of high prevalence in the older population [82,83,84] deserving, therefore, high attention. Other chronic diseases investigated were RA, LSS, spondylolysis, fibromyalgia, IBS, migraine and some local pain such as back, knee, hip, neck and lower-extremity pain. Studies which compared treatment effects on different diseases did not differ according to the number or type of diagnoses [35].

Regarding self-management strategies, a diversity of strategies was used to cover all the biopsychosocial aspects that involve pain. Online and in-person strategies were investigated. Both of them reported mostly positive outcomes. Most studies combined more than one strategy; therefore, it is not possible to affirm that a certain outcome was exclusively due to one of the strategies adopted. For this reason, we opted to report strategies and outcomes in different tables.

The most-investigated strategy was the individual or group self-management course/program, which aims for education and changes in pain and lifestyle and encompasses a mix of most of the other strategies presented in this review. In this sense, some studies also investigated the use of booklets, but this strategy was much less frequent. Both, self-management courses/programs and booklets presented positive results in all three areas of the biopsychological aspects.

Physical exercise was the second most reported strategy used. In fact, this is a “gold standard” intervention, recommended by all guidelines related to painful and chronic conditions [85,86], showing benefits in all biopsychosocial aspects. Exercises specific for the health condition treated demonstrated positive results such as in [32], improved gait [48,52], increased school/work attendance, decreased medication use [53,55], and improved functional capacity and decreased pain-related catastrophizing [55]. General exercises also demonstrated benefits, among them, improved global health [35,63], improved physical function [21,58,59,62], improved lower limb strength, perceived exertion and cognition, aerobic capacity and endurance [36,62], improved self-efficacy for exercise [59], improved communication [36], decreased fatigue [75], increased self-acceptance, self-assertion, hope and engagement coping strategies and increased knowledge in pain [28]. Benefits such as improved ALDs [36,48,54,55], decreased pain [35,36,48,53,54,55,58,59,60,63,75], increased self-care, self-efficacy, SM and/or health behaviors [36,53,54,55,60], improved physical activity level [28,53,75], improved QoL [53,55,62], decreased presenteeism and participation in hobbies [36,55] were found, independent of type of exercise practiced.

To adopt physical exercise as a self-management strategy, the older adult must know how to practice unsupervised exercise in a safe manner, avoiding accidents, and to attempt self-care in posture and movement [31,47,74]. Although many authors have given posture advice, there is no consensus in the literature about causal relationships between physical posture adopted and pain incidence [87]. However, for someone starting to self-manage chronic pain, it is important to know that each type of exercise has a different body effect, to have exercise options, to know some progression strategies, and to recognize when to stop or change exercises. This was also covered by the studies in this review [25,35,36,40]. 

Education and cognitive behavioral therapy (CTB) were both in third place among the self-management strategies used. Studies investigating patient education on pain reported reduction of pain and medication use, improvement of functional capacity [47,74], decrease in daily pain catastrophizing, morning disability, fatigue and anxiety, improvement of pain-related perceived control [64] and not seeking additional care [41], beyond those results reported under self-management courses/programs, which also included educational strategies. To cope with complexity in pain patient care, we must abandon linear models, accept unpredictability, respect (and use) autonomy and creativity, and respond flexibly to emerging patterns and opportunities [88].

Cognitive behavioral therapy (CBT) is a biopsychosocial approach that can be used to encourage patients to star their pain management [6]. In this review, strategies such as meditation/auto-hypnosis, diet, activity pacing, guided physical activity, thought management and self-created coping strategies were covered by CBT, showing positive results for pain [29,55,65,66,67,68], sleep, fatigue [64,65], self-efficacy [39,51,55], catastrophizing, disability [34,51,55,64,68], mood [29,34], depression [29,68], anxiety, perceived control [64], unhelpful pain beliefs ([34,68], functional reach [34], presenteeism, participation in hobbies, school/work attendance, and ADLs [55].

Few studies reported negative results. This was probably due to publication bias [89]. In fact, only three of the studies found in this review dared to publish only neutral or negative results. They reported no difference in the QoL of people who did and who did not adopt exercise, self-management courses and participation in community, and worse QoL for those who changed their diet [77]; no effect of activity pacing on pain, fatigue or physical function [76]; peer-lay led workshops had no effect on pain-related disability or on health expenditure, and positive effects on distress, somatic symptoms, pain catastrophizing and self-efficacy were not maintained after 3 months [50]. The other studies which mentioned neutral or negative results also mentioned positive results for other outcomes [25,36,46,57,60].

The majority of outcomes addressed biological aspects, followed by psychological aspects. However, as most of the studies combined more than one pain management strategy, their outcomes also addressed a mix of biopsychosocial aspects of pain management.

As this study was an integrative review, its limitations are those already reported in the literature for this type of study design. The combination and complexity of incorporating diverse methodologies can contribute to a lack of selection rigor [23]. To reduce these possible effects, study selection was performed by two researchers independently. Methods of analyzing and synthesizing results might have been poorly formulated, as combining empirical and theoretical reports can be difficult, but there is no current guidance on reporting. Therefore, we opted to report the results in two tables: one with the strategies and the other with the main outcomes, as it was not possible to demonstrate which outcome came from a certain strategy. This relationship was, hence, reported in the discussion. As research on this topic for this population is still recent and scarce, the small number of studies of certain strategies and weak methodology of some studies also limited the interpretations. The most common limitations self-reported by the selected studies were a small number of participants and lack of a control group. Future research should focus on the gaps that these limitations potentially created in their conclusions.

As a practical implication, this integrative review highlights the need to encourage older adults to start self-managing their chronic pain symptoms and changing their thoughts and lifestyle. Our results can be used as a guide for health professionals during the creation of educational technologies or while showing their patients how to follow their own path.

## 5. Conclusions

Research on chronic pain self-management for older adults has increased in recent years. Although there are a diversity of chronic physical painful conditions being investigated, many conditions are still under-investigated. Online and in-person strategies are currently adopted, demonstrating similar results. Positive results are evidenced by strategies including health promotion, mind control, social participation and taking action. Major results come from a combination of strategies focusing on biopsychosocial aspects of pain management. Results list not only reduction of pain itself, but increased self-efficacy, adoption of health behaviors and improvement of functionality, among others, i.e., improved QoL, despite pain.

## Figures and Tables

**Table 1:** General topics and contents covered by the main strategies investigated.

Reference		Main Chronic Pain Self-Management Strategies	
	Addressed Field	General Topic	Covered Content
[20,21,30,31,32,33,34,39,41,47,49,51,53,55,56,60,61,62,63,64,65,66,67,68,74]	Health Promotion	Pain education	- anatomy and musculoskeletal biomechanics - causes of pain: biopsychosocial - types of pain - mechanical origin of pain - central sensitization - red flags - common diagnostic strategies - the role of imaging diagnostics - early identification of pain generators - common treatment strategies - use of ice and heat - the role of medication and placebo - the value of exercise and physical activity - evaluation of new treatment possibilities
[25,26,30,31,35,36,39,40,42,43,44,45,48,49,51,52,54,64,65,67,70,73]		Behavior/ Lifestyle	- sleep hygiene - stress, anxiety and depression - leisure scheduling - nutrition - inflammation - physical exercise
[25,28,34,35,42,43,44,45,48,49,51,52,54,55,64,65,66,67,68,69,70,73]	Mind control	Thought management	- negative automatic thoughts - cognitive restructuring - coping thoughts - pleasant images - meditation/self-hypnosis - other distraction techniques - relaxation/breathing - positive reinforcement
[71,78]		Faith, positivity and sharing	- accepting and not complaining, remaining positive - looking for spirituality - motivation and hope - remaining active, engaging in the community - sharing experiences (or not)
[49,75]	Social participation	Lay-peer	- social activities - sharing routines - sharing feelings and techniques to deal with frustration, fears and fatigue - exercise and nutrition experiences - assisting communication with family, friends and health professionals - supporting each other
[20,21,25,32,49,53,56,57,58,59,60,61,62,63,64]	Taking action	Physical exercise	- unsupervised exercise - self-care in posture and movement - specificity of each exercise (aerobic, mobility, stretching, strengthening, balance, explosion, endurance, coordination) - exercise options and progression strategies - recognizing when to stop or change exercises
[19,25,28,29,31,33,34,37,38,42,43,44,45,48,49,51,52,54,55,64,65,66,67,68,70,73]		Self-efficacy	- goals and action planning - decision making - problem solving - ability to deal with pain - graded exposure - stimulation and tolerance - activity–rest rhythm - communication skills - management during the outbreak - monitoring for maintenance/prevention of relapses
[55,57,58,59,65,70,72]		Self-monitoring	- sleep diary - daily activities (type, duration, intensity) - physical exercises performed (feeling of achievement and accumulation of daily effort) - pain diary - diary of thoughts and feelings - thank-you message to yourself for the effort (promote self-care and self-efficacy)

**Table 2:** Positive findings of the studies included.

References	Biopsychosocial Aspects	Examples of Positive Outcomes
[19,20,21,25,26,28,29,30,31,33,34,35,36,37,38,39,40,41,42,43,44,45,47,48,49,51,52,53,54,55,56,57,58,59,60,61,62,63,64,65,66,67,68,69,70,73,74,75]	Biological	- improved ADLs - increased self-care, self-efficacy, SM and/or health behaviors - decreased pain intensity and/or region - improved functional capacity - decreased medication use - decreased disability - decreased fatigue - decreased health care visits - improved physical activity level - improved gait - physical function - decreased swelling, crepitus, limitation of movement and stiffness - reduced disease activity measures - improved sleep - decreased pain interference - reduced claudication - reduced BMI - deepness of breath
[25,26,28,29,30,32,34,35,37,42,43,49,51,53,54,55,64,68,70,71,72,78]	Psychological	- self-created strategies to lead a life, despite pain - decreased pain-related psychological distress - decreased pain-related catastrophizing - decreased daily stress-related anxious affect - decreased daily pain-related perceived control - reduced depression level - decreased fear avoidance - decreased anxiety - improved perceived change - increased pain knowledge - increased satisfaction - increased self-acceptance, self-assertion, hope and engagement coping strategies - decreased unhelpful pain beliefs and improved functional reach - improved mood
[25,36,53,57,59,62,69]	Biopsychological	- improved self-efficacy for exercise - improved lower limb strength, perceived exertion and cognition, aerobic capacity and endurance - decreased desire for surgery - engagement level positively associated with improvements in pain intensity, pain interference and pain self-efficacy - reduced aggravation at rest
[26,36,42,43,49,53,55,71]	Social	- improvement of presenteeism; - participation in hobbies; - increased school/work attendance; - increased vitality and social function - improved communication
[32]	Biosocial	- higher levels of exercises engagement associated with pain decrease
[35,36,38,40,46,53,55,58,62,63,70]	Biopsychosocial	- improved QoL - improved global health

**Table 3:** Main negative and neutral findings of studies included.

Reference	Main Negative or Neutral Outcomes
[25,57]	- no effect on mood or pain-related disability
[77]	- no differences in QoL of people who did and who did not adopt self-management strategies - self-related QoL was worse for those who changed their diet
[36]	- no effect in cognitive symptom management, self-perceived health, lower limb flexibility and handgrip strength
[60]	- no effect for coping beliefs and fear avoidance
[46]	- no change in self-evaluation for most of the participants
[76]	- no effect of activity pacing on pain, fatigue, or physical function
[50]	- disability, distress, somatic symptoms, pain catastrophizing, self-efficacy and health worry reduction were not maintained after 3 months
